# Remote maternal-fetal telemedicine monitoring for high-risk pregnancy care: A feasibility study

**DOI:** 10.1371/journal.pone.0336797

**Published:** 2025-11-14

**Authors:** Jack Le Vance, Alexandra Emms, Victoria Hodgetts Morton, R. Katie Morris, Leo Gurney

**Affiliations:** 1 Department of Applied Health Sciences, School of Health Sciences, College of Medicine and Health, University of Birmingham, Birmingham, United Kingdom; 2 Birmingham Women’s and Children’s NHS Foundation Trust, Birmingham, United Kingdom; Karolinska Institutet, SWEDEN

## Abstract

High-risk pregnancies undergo regular antenatal monitoring, including cardiotocography (CTG) and ultrasound. Recently there has been an emergence of sophisticated remote telehealth interventions, potentially enabling care to be shifted into the home setting. Our aim was to evaluate the feasibility and acceptability of home CTG and home ultrasound monitoring for high-risk pregnancies. This was a single center study. Women aged ≥18 years, English speaking, singleton pregnancy, ≥ 32 weeks gestation and had at least one of four high-risk obstetric conditions were eligible. Participants were randomized to one of three groups: (1) home ultrasound; (2) home CTG; and (3) both, whilst continuing their routine antenatal care. The primary outcome was completion of 20 minutes of interpretable fetal heart recording and/or completion of an interpretable fetal ultrasound for each monitoring episode. Ultrasound interpretability was assessed for three validated criteria: fetal heartbeat, fetal movements and liquor volume assessment. Secondary outcomes included monitoring adherence, anxiety management, acceptability and safety. Fifteen participants, within three groups, completed 24 remote ultrasounds and 59 remote CTGs. Overall, the fetal heartbeat, movements and an assessment of the liquor volume were identified in 92%, 83% and 100% of all ultrasound scans respectively. 79% of all scans had all three criteria unanimously assessed. Three-quarters of all CTGs contained at least 20 minutes of continuous interpretable computerized fetal heartrate recording. Neither ethnicity, parity, BMI nor fetal presentation were significant factors for achievement of the primary outcome for both devices. There was non-significant reduction in anxiety scores before and after device usage (p = 0.19). Participants’ monitoring adherence and acceptability ratings were high in all groups. No adverse maternal-fetal outcomes relating to device usage occurred. Home ultrasound and cardiotocography are potentially feasible and acceptable to high-risk pregnant women. Larger studies are required to refine how best to implement such devices into clinical practice. ClinicalTrials ID: NCT06366711.

## Introduction

Antenatal care is defined as the care delivered to pregnant women to ensure optimal health for both mother and baby [1]. There are several conditions predisposing and arising during pregnancy which classify women as high-risk. High-risk women will undergo additional antenatal monitoring, due to an increased risk of adverse perinatal outcomes [2–4]. Monitoring typically includes regular cardiotocographs (CTGs) for measuring fetal heartrate, regular ultrasound scans to assess biometry and Doppler blood flow, or both.

Strategised monitoring of maternal-fetal well-being within high-risk pregnancies has been a key priority for obstetric care within the United Kingdom (UK). This has arisen from several national initiatives including Saving Mothers and Babies Lives Care Bundle version 3 and MBRRACE-UK [5,6]. Serial CTGs and ultrasounds are core components of this strategised monitoring. Despite stillbirth rates demonstrating an overall decline since 2010, UK figures remain higher than many other counties [5]. Furthermore, literature is cautious on the beneficial impact of antenatal CTG and ultrasound monitoring on reducing adverse perinatal outcomes [7,8].

Regular CTG and ultrasound monitoring requires the mother to attend hospital frequently, which can become a psychological and monetary expense [9,10]. Furthermore, the demand for serial monitoring has surged in recent years, primarily due to guideline changes and an ageing maternal populations, leading to an increased number of women classifying as high-risk. This creates a capacity issue within obstetric services, resulting in appointment delays, which may lead to substandard care [5]. Therefore, discovering pathways to address such concerns is paramount to enhancing the quality of UK obstetric care.

Following the latest pandemic, in an endeavor to reduce face-to-face contact, there was an escalation in telehealth development, including remote monitoring [11,12]. Remote monitoring uses wearable/portable device technology, potentially replacing aspects of outpatient antenatal visits [12]. Studies have demonstrated positive results for remote monitoring with regards to patient experience and safety [13–15]. Recently, more sophisticated technology has emerged, allowing CTG and ultrasound assessments to be conducted remotely, such as in the home setting [13,16–18]. Usage of these devices have become of greater interest in recent times,

as shifting care from the hospital to the home setting aligns closely with the recently published NHS 10 year plan [19].

Consequently, our aim is to explore the feasibility and acceptability of home CTG and ultrasound monitoring for high-risk pregnancies. While a small number of prior studies have explored using either device, our study is unique by investigating the simultaneous use of both technologies [17,18]. To the best of our knowledge, this is the first feasibility study to assess both remote devices within a single patient cohort.

## Methods

### Study population

We conducted a single center feasibility study at the Birmingham Women’s Hospital, Birmingham, UK recruiting between 17^th^ June 2024 and 17^th^ September 2024 (ClinicalTrials ID: NCT06366711). The Birmingham Women’s Hospital delivers approximately 8000 babies per annum, with 72% classifying as high-risk patients.

Eligible participants were approached within the day assessment unit (DAU); a department whereby high-risk patients regularly attend for outpatient CTG and/or ultrasound monitoring. Women aged ≥18 years, fluent in English, with a singleton pregnancy ≥32 weeks gestation and classed as high-risk for one or more of the following reasons were eligible for inclusion: (1) pregnancy induced hypertension; (2) preterm prelabor rupture of membranes; (3) previous history of stillbirth; and (4) obstetric cholestasis. Women additionally had to be prepared to attend hospital on an immediate basis secondary to any unexpected finding detected during home monitoring. Exclusion criteria included: multiple pregnancy, body mass index (BMI) ≥35, women with cardiac devices which may interfere with the CTG device, latex allergies or dermatological conditions that interfere with device placement.

Participants were given a patient information leaflet and provided written informed consent. Consenting participants completed a pre-study questionnaire, which recorded maternal demographics and contained a validated measure of anxiety: Generalized Anxiety Disorder-7 (GAD-7) [20].

### Randomization and blinding

Following consent, participants were randomly assigned to one of three groups: (1) home ultrasound device; (2) home CTG device; and (3) both devices. Participants were randomized using a concealed envelope procedure previously detailed by Torgerson et al [21]. Each envelope contained one of the above three arms. Envelopes were created and sealed by a healthcare professional not involved in the study. The research team then opened envelopes once an eligible participant had consented. Given the nature of the study, neither participant nor the study team were blind to group assignment. No group crossover by participants occurred.

### Procedures

Following randomization, participants had a face-to-face education session on device/s usage to ensure a comprehensive understanding of the equipment. Home ultrasound monitoring was performed using the CE marked Pulsenmore device (GE HealthCare); a device validated for detecting the fetal heartbeat, fetal movements and assessing the liquor volume [22–25]. During the initial encounter, participants downloaded the Pulsenmore smartphone application. The research team would generate a QR code from the online dashboard, which would allow the participants to synchronize their phone, via the application, with the device. The device was compatible for both iPhone and android devices. The research team could limit the number of scans each participant could do to minimize the risk of excess scanning. However, participants were clearly advised to only perform the scan at times jointly agreed between the researcher and participant. Once synchronized, the phone was then inserted into the device cradle to ensure compatibility. Participants were then able to complete home monitoring independently by following a series of five short instructional videos to complete the full scan. Each video consisted of a sweeping motion across different areas of the maternal abdomen, whereby the app videos guided the participant on the correct scan speed, including the adequate probe pressure required to apply on the abdomen. Each complete scan lasted an average duration of three minutes. Following scan completion, the image recordings were uploaded wirelessly for the research team to review on the online dashboard.

Home CTG monitoring was performed using the CE marked PregnaBit Pro device (Nestmedic and ICT Healthcare Technology Solutions). The equipment contained one transducer for fetal heartrate monitoring and one transducer for measuring uterine contractability, secured to the maternal abdomen by elastic bands. The participant would additionally wear a finger pulse oximeter to monitor the maternal heartrate. The transducers and oximeter were connected to the main device via cables and the pulse oximeter was connected via Bluetooth. Participants would be able to record fetal movements via a button on the screen of the device. The research team taught each participant how to set up the equipment and each participant was provided with a step-by-step infographic for home device set-up. Before each monitoring episode, the research team would send a unique pin code to each participant to enable the device to unlock. This was to minimize any risk of monitoring outside of the times agreed between the participant and the research team. The PregnaBit Pro device allowed continuous computerized recording of the fetal heartrate, maternal heartrate, uterine activity and fetal movements. Device data was uploaded remotely and live, minute-by-minute for the research team to review on the online dashboard. If the research team identified that the participant was demonstrating difficulty in maintaining a clear recording of the fetal during the monitoring, the research team would call the participant to help optimize the recording. Following one hour of monitoring, computerized criteria would be provided including a short-term variation. Further information about both devices can be found in S2 File.

Participants performed the ultrasound monitoring fortnightly for up to eight weeks (maximum of four scans per participant) and/or the CTG monitoring twice weekly for up to four weeks (maximum of eight sessions per participant), or until delivery. Participants continued their routine antenatal care, which included antenatal clinic appointments and DAU ultrasound/CTG assessments alongside device usage. The timing of the monitoring was jointly agreed with the participants to maximize compliance and minimize any disturbance with antenatal appointments. Following each monitoring episode participants were called by the research team to discuss the monitoring episode, answer any questions or concerns and schedule the next monitoring episode. Importantly, a strict emergency escalation pathway was implemented in the event of any maternal and/or fetal concern relating to remote device usage. This involved immediate participant attendance to maternity triage for assessment, which included conducting maternal observations and a repeat CTG, as per hospital policy. Following device completion, participants returned the equipment and completed a questionnaire containing multiple choice and free text questions, including a repeat GAD-7 anxiety questionnaire.

### Outcome

We intended to assess the feasibility and acceptability of remote, home ultrasound and CTG monitoring. The primary outcome was completion of 20 minutes of interpretable fetal heartrate recording and/or completion of an interpretable fetal ultrasound scan for each monitoring episode. Ultrasound interpretability was assessed against the three validated criteria: fetal heartbeat, fetal movements and assessment of the liquor volume. To reduce observer bias, two researchers (JLV and AE) independently interpreted the ultrasound recordings. If there was a difference in judgement a third assessor (LG) independently reviewed the recordings, and a final assessment was determined. The researchers were able to quantify the liquor volume on the scan recordings within the online dashboard. The maximum pool depth (MPD) was determined from the recordings and measured using the caliper measurement function. Ultrasound recordings were also assessed for fetal presentation by two independent researchers; a criterion the device is currently not validated for. Remote ultrasound scans performed within seven days of a departmental hospital scan were correlated against each other to determine comparability of liquor volume measurements and fetal presentation. Remote and hospital performed liquor volume measurements were classified as either normal (MPD 3–8 cm) or polyhydramnios (MPD > 8 cm) and compared categorically, rather than specific variations between liquor measurements. This was due to small variations in third trimester liquor measurements seen over seven days [26].

Secondary outcomes included monitoring adherence, defined as the difference between the scheduled time to start monitoring and subsequent commencement, participant anxiety, participant acceptability and device safety. Anxiety management was assessed using the GAD-7 questionnaire, whereby seven questions contributed to a total score out of 21 [20]. Lower scores indicated milder anxiety. Questionnaires assessed participant acceptability and were categorized into topics such as device monitoring, reassurance, responsibility and future considerations. Questions were asked on a 5-point Linkert scale, with two additional free text questions. Questions were developed and informed by the validated theoretical framework of acceptability by Sekhon et al [27]. Device safety was determined by the number of hospital attendances secondary to remote device usage, including any adverse maternal-fetal outcomes directly related to study involvement.

### Statistical analysis

Raw data was reported as frequencies, either as mean and standard deviation or median and interquartile range (IQR). Differences between two groups was compared using the Student’s t-test for continuous parametric variables and the Mann- Whitney U test for non-parametric variables. Categorical variables between two groups were compared using the Fisher Exact test. Anxiety scores were analyzed using the Wilcoxon matched-pair test. Graphical analyses of anxiety scores were performed using GraphPad PRISM 7.05 (GraphPad Software, La Jolla, CA, USA). A P value of <0.05 was statistically significant. To detect a 30% difference in pre- and post-study anxiety scores, at a power of 90% and alpha at 0.05, a minimum sample size of 14 was required. Fifteen participants were recruited to allow an even split between all groups.

### Patient and public involvement

A Patient and Public Involvement and Engagement group of five ethnically diverse women, comprising of high-risk obstetric conditions helped co-design all participant-facing materials and inform on study design.

### Ethical approval

The study was ethically approved by the East Midlands – Leicester Research Ethics Committee (REC: 24/EM/0052) on 28^th^ March 2024 and the Health Research Authority (IRAS332918).

## Results

**Fig 1:**
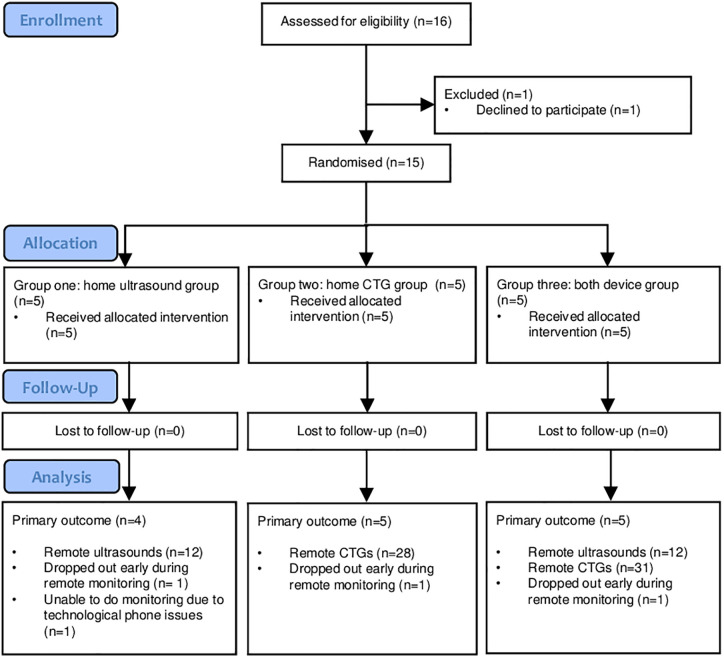
CONSORT flowchart.

Between May 2024 and September 2024, 16 women were approached to recruit 15 participants, demonstrating a recruitment rate of 94%, who were randomly assigned to one of the three groups (Fig 1). No participants were lost to follow-up. Group demographics can be seen in Table 1 [28]. The most common conditions for enrolment were obstetric cholestasis (40%), previous history of stillbirth (27%) and pregnancy induced hypertension (27%). There were no differences in baseline characteristics between groups. The CONSORT checklist can be seen in S1 File.

**Table 1:** Baseline characteristics.

	Group One: USS only (n = 5)	Group Two: CTG Only (n = 5)	Group Three: Both devices (n = 5)
Age (mean (SD))	32 (6.4)	27.6 (2.8)	28.4(5.9)
Gravida (median (IQR))	3 (2 –4 )	3(1 –3)	3 (2 –3 )
Parity (median (IQR))	1 (0-2)	0(0-1)	2(1 –2)
BMI, kg/m^2^ (mean (SD))	28.6(4.7)	30.8(2.0)	27.6(3.5)
18.5–24.9	1 (20%)	0 (0%)	1 (20%)
25.0–29.9	2 (40%)	2 (40%)	3 (60%)
30.0–34.9	2 (40%)	3 (60%)	1 (20%)
White British	1 (20%)	3 (60%)	3 (60%)
Asian	2 (40%)	2 (40%)	1 (20%)
African	1 (20%)	0 (0%)	0 (0%)
Other	1 (20%)	0 (0%)	1 (20%)
Level 2	0 (0%)	1 (20%)	1 (20%)
Level 3	0 (0%)	2 (40%)	2 (40%)
Level 4	0 (0%)	0 (0%)	0 (0%)
Level 5	1 (20%)	0 (0%)	0 (0%)
Level 6	2 (40%)	2 (40%)	2 (40%)
Level 7	2 (40%)		
Distance from home to hospital, miles (mean (SD))	4.3 (1.8)	6.2 (2.7)	4.9 (1.4)
Pregnancy induced hypertension	2 (40%)	2 (40%)	0 (0%)
Obstetric cholestasis	1 (20%)	2 (40%)	3 (60%)
Previous history of stillbirth	2 (40%)	1 (20%)	1 (20%)
Preterm prelabour rupture of membranes	0 (0%)	0 (0%)	1 (20%)
Current smoker	0	1	1
Low Papp-A	1	0	0
Gestational diabetes	0	1	1
Thyroid disease	0	1	0
Previous preterm birth	1	0	3
Previous pre-eclampsia	1	0	1
Previous SGA	0	1	2
Gestational age at start of monitoring, weeks (median (IQR))	33 + 0 (32 + 3-34 + 3)	33 + 2 (32 + 2-34 + 2)	33 + 0 (32 + 0-33 + 3)
Time for consent, enrolment and educational session, minutes (mean (SD))	37.4 (14.2)	33.2 (5.1)	47.6 (13)
Duration of enrolment, days (mean (SD))	28 (10)	17 (7)	24 (12)

BMI, body mass index; CTG, cardiotocography; IQR, interquartile range; Papp-A, pregnancy associated plasma protein A; SD standard deviation; SGA, small for gestational age; USS, ultrasound; WHO, world health organisation

*Educational status based on UK government’s education level system ranging from entry to level 8 [28]. Entry level: entry level award or equivalent, Level 1: GCSE (grades 1–3) or equivalent, Level 2: GCSE (grades 4–9) or equivalent, Level 3: A level or equivalent, Level 4: certificate of higher education or equivalent, Level 5: diploma of higher education or equivalent, Level 6: degree with honors or equivalent, Level 7: master’s degree or equivalent, Level 8: doctorate or equivalent.

### Remote ultrasound monitoring

Twenty-four remote ultrasound scans were completed by nine participants, evenly split between the first and third groups. Eight scans were completed prior to 33 + 6, seven scans between 34 + 0–35 + 6, six scans between 36 + 0–37 + 6 and three scans after 38 + 0 weeks gestation. The overall median number (IQR) of scans completed between women was 3 (1–4). One woman within group one could not complete any scans due to phone techno-compatibility issues. One woman within group three completed just one scan as her phone broke following enrolment. The median number of scans within groups one and three were 3(1–4) and 2(1–4) respectively. Overall women demonstrated good adherence to performing remote ultrasound monitoring at the scheduled times, with a median of 11 minutes (3–17 minutes) between the scheduled time and scan commencement. Median time within group one versus group three was slightly less, however, not significant (5 minutes (3–46 minutes) versus 12 minutes (7–16 minutes), p = 0.20). The median time to analyze each ultrasound scan by a single reviewer following participant completion (including time for scan to upload on to the online platform) was 34 minutes (28–48 minutes). There was no difference between groups; group one, 41 minutes (30–68 minutes) versus group three, 31 minutes (23–45 minutes) (p = 0.53).

Overall, the fetal heartbeat, movements and an assessment of the liquor volume were identified within 92%, 83% and 100% of all scans respectively. In total, 79% of all scans had all three criteria unanimously assessed, attributed to 75% of group one scans versus 83% of group three scans. Comparing all ultrasounds completed within the first two scheduled episodes versus the final two completed episodes, there was no significant difference in achieving all three criteria (75% versus 88%, p = 0.63). All remote ultrasounds were analyzed for fetal presentation, as aspect that women were not trained to do, and the research team independently evaluated. Three scans were uninterpretable for presentation, all occurring after 37 weeks gestation. Sixteen ultrasounds had a corresponding departmental ultrasound within seven days of the remote scan, whereby 88% correlated with the same presentation. Half of scans were correctly assessed after 34 weeks gestation.

**Fig 2:**
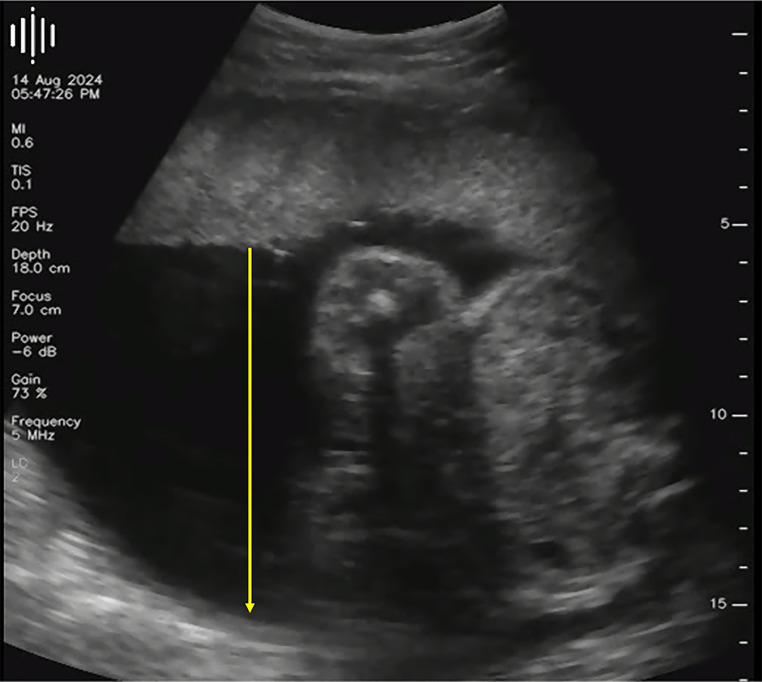
Participant using remote ultrasound device and demonstrating polyhydramnios measuring a maximum pool depth of 9.7 cm (yellow arrow).

All ultrasound scans were measurable for liquor volume, with a mean MPD of 6.6 cm ± 1.9 cm. Five (21%) remote ultrasound scans measured above 8 cm, classifying as polyhydramnios (Fig 2). Four of these five remote scans had an outpatient hospital scan within seven days, of which 75% correspondingly demonstrated polyhydramnios. The remote scans occurred at 32 weeks, 33 + 2 weeks and 35 weeks gestation. Conversely, 19 (79%) remote scans demonstrated normal liquor volumes, of which 14 had a departmental scan within seven days, whereby 93% similarly displayed normal liquor volumes. Eight of these corresponding scans occurred after 34 weeks gestation.

Neither parity, ethnicity, educational status, BMI nor fetal presentation were significant factors for completion of a remote scan with respect to achieving all three criterion (Table 2). One participant in either group exited the study early, due to external reasons. All other participants completed either the maximum number of scans attainable or until delivery. There were no maternal-fetal adverse outcomes relating to remote ultrasound scan usage and no participants required referral to maternity triage.

**Table 2:** Comparison of demographic factors with regards to competency of scan completion.

		All three ultrasound criteria seen n (%)	Fewer than three ultrasound criteria seen n (%)	P Value
Parity	0	7 (88%)	1 (12%)	0.63
	≥1	12 (75%)	4 (25%)	
Ethnicity	White British	11 (92%)	1 (8%)	0.32
	Non-White British	8 (75%)	4 25%)	
Educational status	Education level ≤5	8 (73%)	3 (27%)	0.63
	Education level >5	11 (85%)	2 (15%)	
WHO classification of body mass index	30.0–34.9	11 (79%)	3 (21%)	0.64
	25.0–29.9	6 (67%)	3 (33%)	
Presentation of fetus	Cephalic	16 (76%)	5 (24%)	1.00
	Non cephalic	3 (100%)	0 (0%)	

WHO, world health organisation.

### Remote cardiotocography monitoring

Fifty-nine remote CTGs were completed by ten participants, evenly split between groups two and three. 56 (95%) CTGs were reviewed live, minute-by-minute, as the patient was monitoring. Overall, the median number (IQR) of CTGs completed per participant was 7 (5–8). The median number of CTGs between groups two and three were 6(4–8) and 8(4–8). Three CTGs did not complete the full hour; one due to insufficient battery charge, one due to maternal illness and another due to a technical fault. All CTGs were repeated the same day, completing the full hour and replacing the index CTG in subsequent analysis. Overall, participants demonstrated a very good adherence to performing the monitoring at the scheduled times, with a median of 5 minutes (2–10 minutes) between the scheduled time and monitoring commencement. Median time between within group two versus group three was slightly less, however, not significant at 4 minutes (1–7 minutes) versus 7 minutes (2–28 minutes) (p = 0.20).

**Fig 3:**
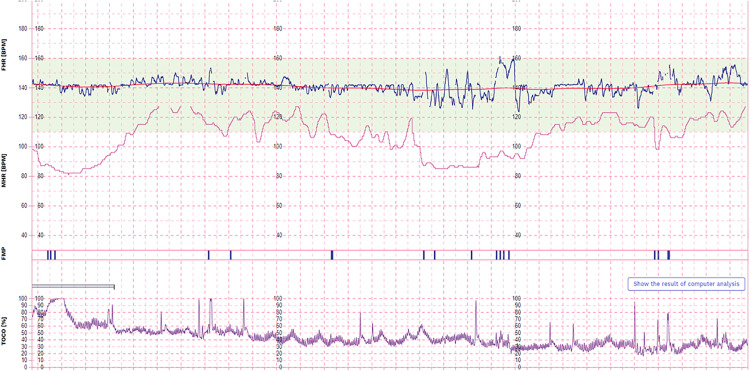
A 20-minute period of remote CTG monitoring performed by one participant, depicting the fetal heartrate (blue line), maternal heartrate (pink line), uterine activity (purple line) and average fetal baseline (red line).

Regarding the primary outcome, 75% of all CTGs (n = 44) contained at least 20 minutes of continuous interpretable fetal heartrate recording (Fig 3). When stratifying between groups, 81% (n = 25) of CTG episodes achieved the primary outcome within group three versus 68% (n = 19) in group two (p = 0.37). Comparing CTGs completed within the first four scheduled episodes versus the final four episodes, there was no difference in monitoring competency (75% versus 74%). Across the full hour, 42% of CTGs (n = 25) contained more than 30% loss of contact, either continuously or sporadically throughout. This was evenly distributed between groups. All participants required at least one phone call from the research team across all monitoring episodes, primarily to assist in improving the fetal heart recording. Eight CTG episodes did not have a calculated short-term variation due to extensive loss of contact. A single participant attributed five of the eight episodes.

Neither parity, ethnicity, BMI, fetal presentation nor placental position were significant factors for achievement of the primary outcome (Table 3). A lower participant educational level demonstrated an improved ability to perform CTG monitoring (p = 0.01), however, this disparity was attributed to a single participant in group two who had several episodes of insufficient monitoring.

**Table 3:** Comparison of demographic and antenatal factors in relation to cardiotocography competency with respect to the primary outcome.

		Interpretable 20 minutes of CTG	Non-interpretable 20 minutes of CTG	P Value
Parity	0	13 (81%)	3 (19%)	0.74
	≥1	31 (72%)	12 (28%)	
Ethnicity	White British	34 (81%)	8 (19%)	0.10
	Non-White British	10 (59%)	7 (41%)	
Educational status	Education level ≤5	32 (86%)	5 (14%)	**0.01**
	Education level >5	12 (55%)	10 (45%)	
WHO classification of body mass index	30.0–34.9	16 (70%)	7 (30%)	1.00
	25.0–29.9	20 (71%)	8 (29%)	
Presentation of fetus	Cephalic	32 (71%)	13 (29%)	0.48
	Non cephalic	12 (86%)	2 (14%)	
Placental position	Anterior	29 (74%)	10 (26%)	1.00
	Posterior	15 (75%)	5 (25%)	

CTG, cardiotocography; WHO, world health organisation.

Eight (14%) CTG episodes required referral for hospital assessment. Five due to a short-term variation <5, one due to a raised fetal baseline, one due to fetal decelerations and one for maternal tachycardia. All were assessed within maternity triage the same day and underwent a repeat CTG, all demonstrating normality. One participant in each group exited the study early, due to external reasons. All other participants completed either the maximum number of CTGs attainable or until delivery. There were no maternal-fetal adverse outcomes relating to remote CTG usage.

**Fig 4:**
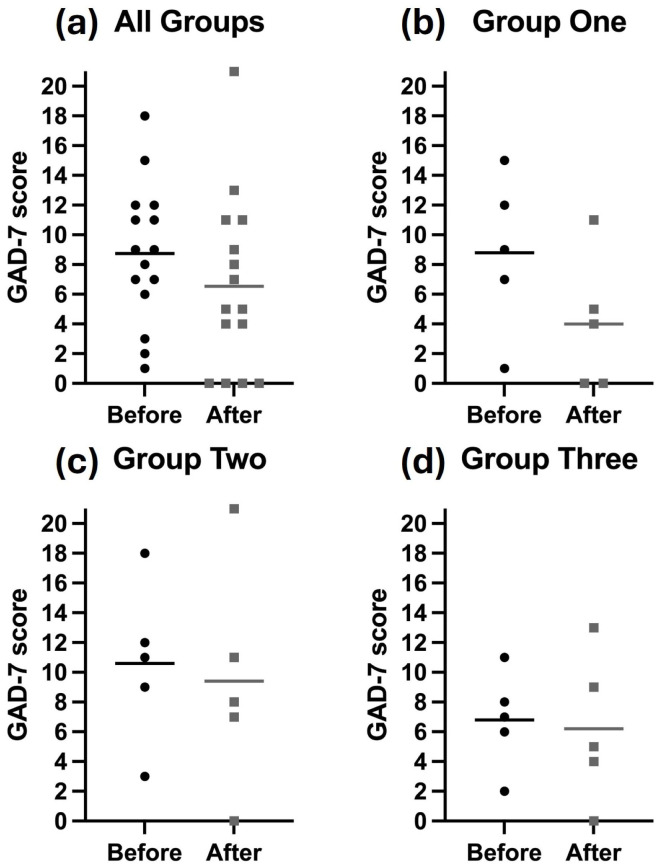
Graphs reporting analysis of maternal anxiety assessed using the GAD-7 anxiety questionnaire. (a) All groups, (b) group one using ultrasound device only, (c) group two using cardiotocography device only, (d) group three using both devices.

There was no statistically significant reduction in anxiety scores prior to and following the usage of all remote devices (mean 8.7 versus 6.5, p = 0.19) (Fig 4).

Ninety percent, 100% and 100% of participants using the ultrasound device either strongly agreed or agreed that the device was easy to use, easy to set up and comfortable. Response rates for the CTG device were 90%, 90% and 80% respectively. Questionnaire responses are within Table 4. Participants cited benefits of home monitoring with regard to maternal reassurance, real-time feedback, increased antenatal understanding, facilitating maternal control, convenience and reduced economic burden. Some participants raised concerns relating to technological barriers and difficulty acquiring the fetal heartrate. Despite this, following study completion, 73% of all participants strongly agreed or agreed that they would be happy to solely receive home monitoring rather than hospital monitoring within a subsequent pregnancy, if safe to do so; increased from 40% prior to device usage.

**Table 4:** Questionnaire responses from participants. Number of respondents shown with proportion in parentheses.

Statement		Strongly Agree	Agree	Neutral	Disagree	Strongly Disagree
**Device monitoring**						
Home monitoring improved my access to care	USS device	3 (30)	5 (50)	2 (20)	0 (0)	0 (0)
	CTG device	3 (30)	5 (50)	2 (20)	0 (0)	0 (0)
Home monitoring allowed me to spend more time with my loved ones	USS device	2 (20)	4 (40)	4 (40)	0 (0)	0 (0)
	CTG device	2 (20)	2 (20)	5 (50)	1 (20)	0 (0)
Home monitoring reduced the level of disruption to my life compared to hospital monitoring	USS device	2 (20)	4 (40)	1 (10)	3 (30)	0 (0)
	CTG device	0 (0)	4 (40)	3 (30)	3 (30)	0 (0)
Overall, I found home monitoring was a convenient method to check my baby’s wellbeing	USS device	4 (40)	5 (50)	1 (10)	0 (0)	0 (0)
	CTG device	7 (70)	2 (20)	1 (10)	0 (0)	0 (0)
**Reassurance**						
I felt safe when undergoing home monitoring	USS device	4 (40)	5 (50)	1 (10)	0 (0)	0 (0)
	CTG device	4 (40)	5 (50)	1 (10)	0 (0)	0 (0)
I felt reassured of my baby’s wellbeing when I performed my own home monitoring	USS device	6 (60)	2 (20)	2 (20)	0 (0)	0 (0)
	CTG device	6 (60)	2 (20)	2 (20)	0 (0)	0 (0)
I felt anxious not having hospital staff close by	USS device	0 (0)	1 (10)	4 (40)	2 (20)	3 (30)
	CTG device	0 (0)	2 (20)	3 (30)	1 (10)	4 (40)
**Responsibility and control**						
I felt like I had more control over my care and my baby’s wellbeing whilst monitoring at home	USS device	3 (30)	4 (40)	2 (20)	1 (10)	0 (0)
	CTG device	5 (50)	3 (30)	1 (10)	1 (10)	0 (0)
I felt that I know more about my baby’s wellbeing whilst undergoing home monitoring	USS device	3 (30)	5 (50)	1 (10)	1 (10)	0 (0)
	CTG device	6 (60)	2 (20)	1 (10)	1 (10)	0 (0)
**Overall**						
Overall, I am satisfied with my experience using this device	USS device	7 (70)	3 (30)	0 (0)	0 (0)	0 (0)
	CTG device	6 (60)	4 (40)	0 (0)	0 (0)	0 (0)
**Future**						
I would be happy to only have home monitoring appointments rather than hospital appointment during my next pregnancy, if it was safe for me and baby	Pre-study	4 (27)	2 (13)	5 (33)	4 (27)	0 (0)
	Post-study	5 (33)	6 (40)	0 (0)	1 (7)	3 (20)
I would feel reassured to only have home monitoring in my next pregnancy if a midwife and/or doctor discussed the results with me on the same day	Pre-study	3 (20)	2 (13)	6 (40)	4 (27)	0 (0)
	Post-study	6 (40)	4 (27)	2 (13)	1 (7)	2 (13)
I feel that my expenses would be less if I were to only have home monitoring during my next pregnancy rather than hospital appointments	Pre-study	5 (33)	4 (27)	5 (33)	0 (0)	1 (7)
	Post-study	8 (53)	5 (33)	1 (7)	0 (0)	1 (7)

CTG, cardiotocography; USS, ultrasound.

## Discussion

This prospective study demonstrates that the use of home ultrasound and CTG monitoring is potentially feasible and acceptable to high-risk women. A high proportion of ultrasound and CTG episodes achieved the primary outcomes and were clinically interpretable. Participants demonstrated good adherence to device usage, with monitoring competency unaffected by demographical characteristics, such as education status, parity and ethnicity and for BMI < 35. Stratification between the first and second halves of monitoring episodes in either device demonstrated no difference in competency, suggesting quick device learning curves. Furthermore, the ultrasound device also demonstrated a potential application for determining fetal presentation; an aspect not yet extensively assessed. Importantly, 95% of CTG episodes were reviewed in real-time, with abnormal traces swiftly identified and reassessed within hospital. Participants demonstrated a high acceptability for both devices, with a trend towards a reduction in anxiety scores. Importantly, no adverse outcomes occurred secondary to device usage.

The utilization of remote telemonitoring has become an important consideration following the recent pandemic, with several obstetric services endeavoring to enhance the patient’s antenatal journey, streamline care and optimize healthcare costs. The recent HoTeL trial demonstrated that daily home CTG monitoring is non-inferior to inpatient CTG monitoring with regards to adverse perinatal outcomes of high-risk pregnancies, whilst reducing mean antenatal cost by 18% per patient [17]. A qualitative assessment by the same team further demonstrated that home monitoring may aid with anxiety relief compared to hospital admission [29]. Several other studies have also investigated the feasibility of remote fetal monitoring, demonstrating positive results [16,30–32]. A multi-site study of 101 low-risk women, comprising of 1278 remote CTG recordings revealed that 99.8% of episodes contained at least 10 minutes of continuous monitoring, alongside a high participant satisfaction [32]. Current literature, including our study, exhibited the potential beneficial applications for remote CTG monitoring within many obstetric populations. Given the ever-increasing number of high-risk obstetric patients within the UK specifically, and the capacity issues this poses on obstetric services, remote CTG monitoring may pose a partial remedy for these concerns. A UK multisite randomized controlled trial (RCT) evaluating remote CTG monitoring as a supplement against routine outpatient management may potentially reshape how care is delivered to pregnant women.

The advantageous use of home ultrasound has also been recently revealed within the literature. Hadar et al reported a high detection rate for fetal movements, heartbeat and normal liquor volume assessment within 1360 remote scans performed by 100 women, including a highly rated user experience [18]. Furthermore, a recent RCT of 50 women with a history of recurrent miscarriage demonstrated a significant reduction in anxiety scores using home ultrasound compared with conventional care [33]. Literature has represented home ultrasound monitoring as a potentially feasible and acceptable method for a basic assessment of fetal wellbeing [22–25]. However, at present our study was unable to identify the fetal heartbeat in all remotely performed scans, which is an essential requirement for any fetal ultrasound scan conducted antenatally to assess the most basic aspects of fetal wellbeing. This initially limits the comparability of a remote scan to what is already conducted within the hospital setting. Further improvements in scan quality and increasing the duration of the scan may improve the identification of certain fetal parameters. Furthermore, for widespread clinical implementation, it is pertinent for the device technology to perform tests analogous to tests currently conducted within a hospital setting. The integration of an umbilical artery Doppler via artificial intelligence would enable direct comparability to hospital performed scans and could have grounds for an impactful device that potentially transforms how antenatal care is currently delivered. We additionally assessed the application of this device for determining fetal presentation; novel notion to consider given the recently funded UK study examining community handheld point of care ultrasound for fetal presentation [34]. However, the clinical utility of home fetal presentation assessment, particularly prior to the onset of labor may be of limited future value if used for only this purpose.

Remote monitoring has the potential to foster greater patient autonomy, whilst reducing psychological strain [33]. Wider implementation of remote monitoring may aid in streamlining outpatient services, potentially providing economic benefit [17,35]. Importantly, home monitoring facilitates the engagement of women more likely to not routinely seek hospital care, particularly those known to suffer health inequalities, such as women of ethnic minority and low socioeconomic backgrounds [5]. It is vital to ensure that the safety of women performing home monitoring is adequately managed.

Strengths of this study include the participant randomization, a broad diversification of participants, a high number of real-time CTG reviews and a well-executed safety netting protocol. This is the first feasibility study to jointly assess both devices within a high-risk pregnancy cohort. A small sample size represents the main study limitation. It may be pertinent to expand on the inclusion criteria, ensuring to demonstrate usage of the remote CTG device in high-risk conditions with overtly placental mediated conditions, such as fetal growth restriction. Furthermore, a small sample size limited the ability to deeply assess and validate a wide variation of liquor volumes using the home ultrasound device. Consequently, we were unable to assess the precision of the device in cases complicated by oligohydramnios, which would be clinically useful given the association of reduced liquor volume and placental dysfunction. Additionally, recruitment was restricted due to device licensing limitations relating to gestational age and BMI. Further studies may want to trial this technology in earlier gestations and larger BMIs (≥35) to determine feasibility of monitoring and if it is possible to widen the current capabilities of these home monitoring devices. However, it should be noted that Doppler technology can be inhibited in high BMI patients, as frequently seen in hospital CTG and ultrasound scans. Furthermore, all participants using the CTG device required at least one phone call from the research team during all monitoring episodes to help with improving the fetal heartrate recording. Enhanced communication within a research setting limits the direct transferability of these devices into the clinical setting. Improvements in face-to-face device education at the time of enrolment may help with improvements in independent remote fetal monitoring. If such devices are used in clinical settings there may be a requirement for dedicated clinical teams who are able to review each monitoring episode at the time of completion. An economic evaluation would be required to determine if this were financially suitable and should be balanced with other potential cost saving aspects of home monitoring such as reduced hospital attendance. This should be assessed within the format of a clinical trial in the first instance. Alternatively, non-invasive fetal electrocardiography (NIFECG) is an alternative modality to consider and has demonstrated advantages to CTG with respect to fetal-maternal heartrate separation, including improved signal acquisition in raised BMIs [36]. This usage of home NIFECG is emerging in the literature, demonstrating a promising assessment for remote fetal well-being. However, the presence of vernix caseosa is hypothesized to reduce signal conductance secondary to insulation, resulting in poor detection of fetal QRS complexes [36]. Vitally, this requires addressing prior to evaluation on a wider scale, however, it is an important technology to further consider for home fetal monitoring. Additionally, we were unable to assess the usage of these devices in those not fluent in English. However, usage of video and transcribed material may help support the inclusion of additional patient groups. Finally, the timing and type of anxiety questionnaire used within this study may require optimization to clearly reflect the impact of remote monitoring on anxiety management. Using the Pregnancy Specific Anxiety scale before and after several individual monitoring episodes may be more impactful rather than a general anxiety score used at study start and end [37].

## Conclusion

This study demonstrates that home ultrasound and home cardiotocography is potentially feasible and acceptable to high-risk pregnant women. In an era of rapid digital health innovation, home monitoring may play a key role in shaping the future of obstetric antenatal care. Larger studies are required to refine how best to implement such devices within clinical practice and determine if they can be widely adopted to an extensive range of women.

## Supporting information

S1 File CONSORT Checklist. (PDF)

S2 File Device information. (PDF)
