# Supplementary material for: Remote maternal-fetal telemedicine monitoring for high-risk pregnancy care: A feasibility study
Source: PLoS One. 2025 Nov 14;20(11):e0336797. doi: 10.1371/journal.pone.0336797 (PMC12617905; doi:10.1371/journal.pone.0336797)
Supplement: S2 File — (PDF) [file pone.0336797.s002.pdf]

Device information

PregnaBit Pro Device (Nestmedic, Poland)

Information below is supplied from the device booklet.

Pregnabit Pro is a CE marked medical device for telediagnosis that supports physicians and midwives who watch over a pregnancy. The CTG is of the same quality as one performed with a stationary cardiotocograph, subject to specialist's evaluation and providing the pregnant patient with recommendations for further management.

The Pregnabit Pro device is used to monitor a pregnancy anywhere and anytime without the need for hospitalization and/or frequent visits to a specialist Clinic, Health Facility or Emergency Room. Each recording is evaluated by the specialists at the telemedical platform. With a Pregnabit Pro device, a teleCTG can be performed on a pregnant woman in her third trimester or earlier, especially when quick access to a specialist is difficult and the pregnancy requires closer monitoring.

The Pregnabit Pro device examines fetal heart rate, uterine muscle contraction activity, maternal heart rate and registers fetal movements felt by the mother. The mother can set up the device following a series of step-by-step instructions. The data is sent continuously by data transmission in connection with a mobile network. When the examination is completed, a CTG recording is sent directly from the device to be interpreted by qualified medical staff via the PregnaOne platform.

The Pregnabit Pro device determines fetal heart rate from 70 to 240 beats/min, with an accuracy of ±4 beats/min. The maternal heart rate range is 25 to 250 beats/min, with an accuracy of ±2 beats/min. The sensitivity of detecting maternal and fetal heart rate coincidence is 84.1%, calculated by Nestmedic.

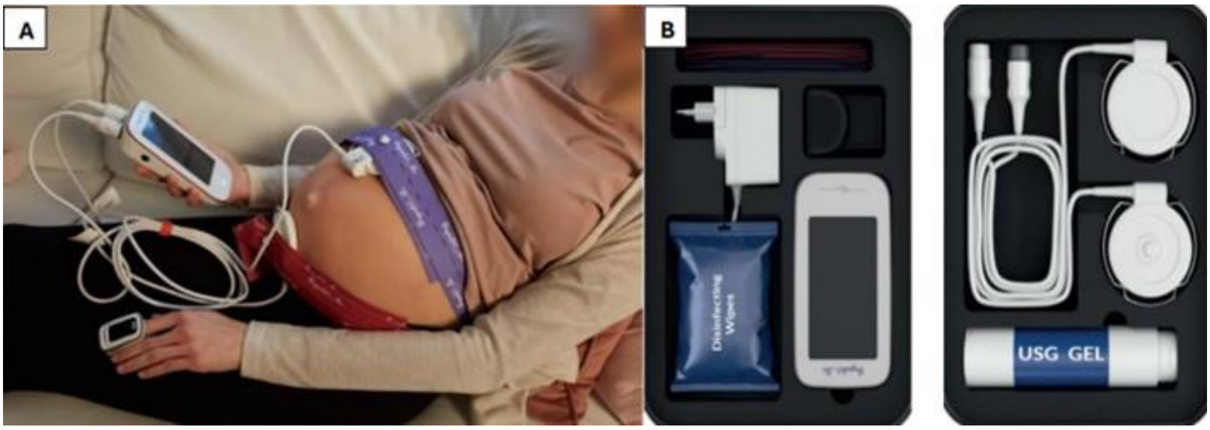

Figure S1: (A) Women using the device at home. The photo was provided by the Nestmedic S.A. (B) Device case with contents. Includes Pregnabit Pro device, two transducers, two abdominal belts, charging cable, pulse oximeter, ultrasound gel and disinfectant wipes. Permission for image use received from Nestmedic.

Pulsenmore ES Ultrasound Device (GE Healthcare, USA)

Information below is supplied from the device booklet.

The Pulsenmore ES ultrasound system consists of an ultrasound device, a mobile application, and the Clinician Dashboard. The device captures the ultrasound images and transfers them to the Clinician Dashboard via a designated mobile app. The Pulsenmore ES ultrasound system is designed to enable the healthcare professional to review fetus ultrasound scans performed in the comfort of your home.

The Pulsenmore ES ultrasound device is designed for single patient use, to prevent cross contamination. Once the Pulsenmore™ app is installed on your smartphone, you must have a Key from your clinician to start a scan. The Key will be provided as a QR code or a link. The duration of each scan is limited (i.e. up to 3 minutes in an App-Guided scan or at the clinician's discretion in a Clinician Guided scan). All users will go through the same tutorial steps for an App-Guided scan. Audio-video tutorials for the 5 steps are provided for this mode only. The Skin Contact Indicator is enabled during an App-Guided scan and Clinician-Guided scan. When this feature is enabled, the app will alert you if skin contact with part of the device's transducer is not optimal. After completing all five (5) scan steps, the videos of the ultrasound scan will be automatically uploaded and sent to a clinician for review.

The Pulsenmore ES ultrasound system utilizes internet connection for uploading scans. The system provides only 2D imaging (B mode) which provides anatomical imaging. The system supports only low frequency (2-5MHz).

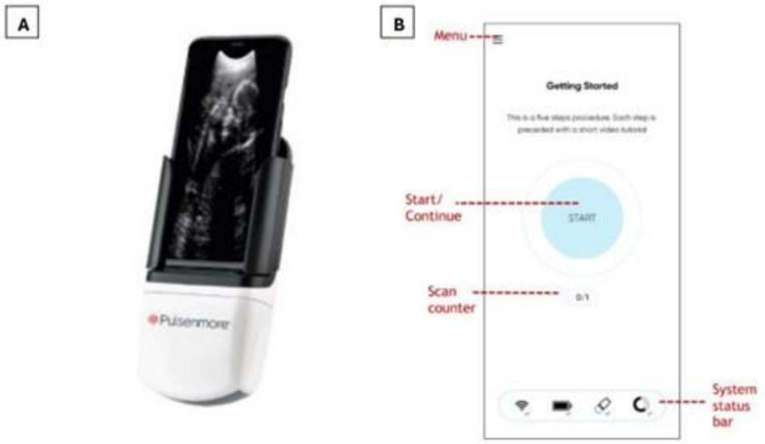

Figure S2: (A) Pulsenmore device (B) Display screen on app that women will view prior to scanning on the Pulsenmore app. Permission for image use received by GE Healthcare.
